# Genome analysis identifies the mutant genes for common industrial Silverblue and Hedlund white coat colours in American mink

**DOI:** 10.1038/s41598-019-40918-7

**Published:** 2019-03-14

**Authors:** Andrey D. Manakhov, Tatiana V. Andreeva, Oleg V. Trapezov, Nikolay A. Kolchanov, Evgeny I. Rogaev

**Affiliations:** 1 Department of Genomics and Human Genetics, Vavilov Institute of General Genetics, Russian Academy of Sciences, Moscow, 119991 Russia; 2 Center for Genetics and Genetic Technologies, Faculty of Biology, Lomonosov Moscow State University, Moscow, 119234 Russia; 3 Center for Brain Neurobiology and Neurogenetics, Institute of Cytology and Genetics, Siberian Branch of the Russian Academy of Sciences, Novosibirsk, 630090 Russia; 4 Department of Animals and Human Genetics, Institute of Cytology and Genetics, Siberian Branch of the Russian Academy of Sciences, Novosibirsk, 630090 Russia; 5 Novosibirsk State University, Novosibirsk, 630090 Russia; 6 Systems Biology Department, Institute of Cytology and Genetics, Siberian Branch of the Russian Academy of Sciences, Novosibirsk, 630090 Russia; 7 Department of Psychiatry, University of Massachusetts Medical School, Worcester, MA 01604 USA

## Abstract

The fur colour of American mink (*Neovison vison*) involves over 35 traits, but only three of these have been linked to specific genes. Despite being the most popular, coat colours Silverblue and Hedlund white remain uncharacterized genetically. The former is the first genetic mutant of fur colour identified in minks, while the latter is a commercially valuable phenotype that can be dyed easily. Here, we performed the whole genome sequencing for two American mink breeds with Silverblue and Hedlund white coats. We identified mutations in splice donor sites of genes coding melanophilin (*MLPH*) and microphthalmia-associated transcription factor (*MITF*) that regulate melanosome transport and neural-crest-derived melanocyte development, respectively. Both mutations cause mRNA splicing impairments that lead to a shift in open reading frames of MLPH and MITF. We conclude that our data should be useful for tracking economically valuable fur traits in mink breeding programs to contribute to global fur production.

## Introduction

**Figure 1:**
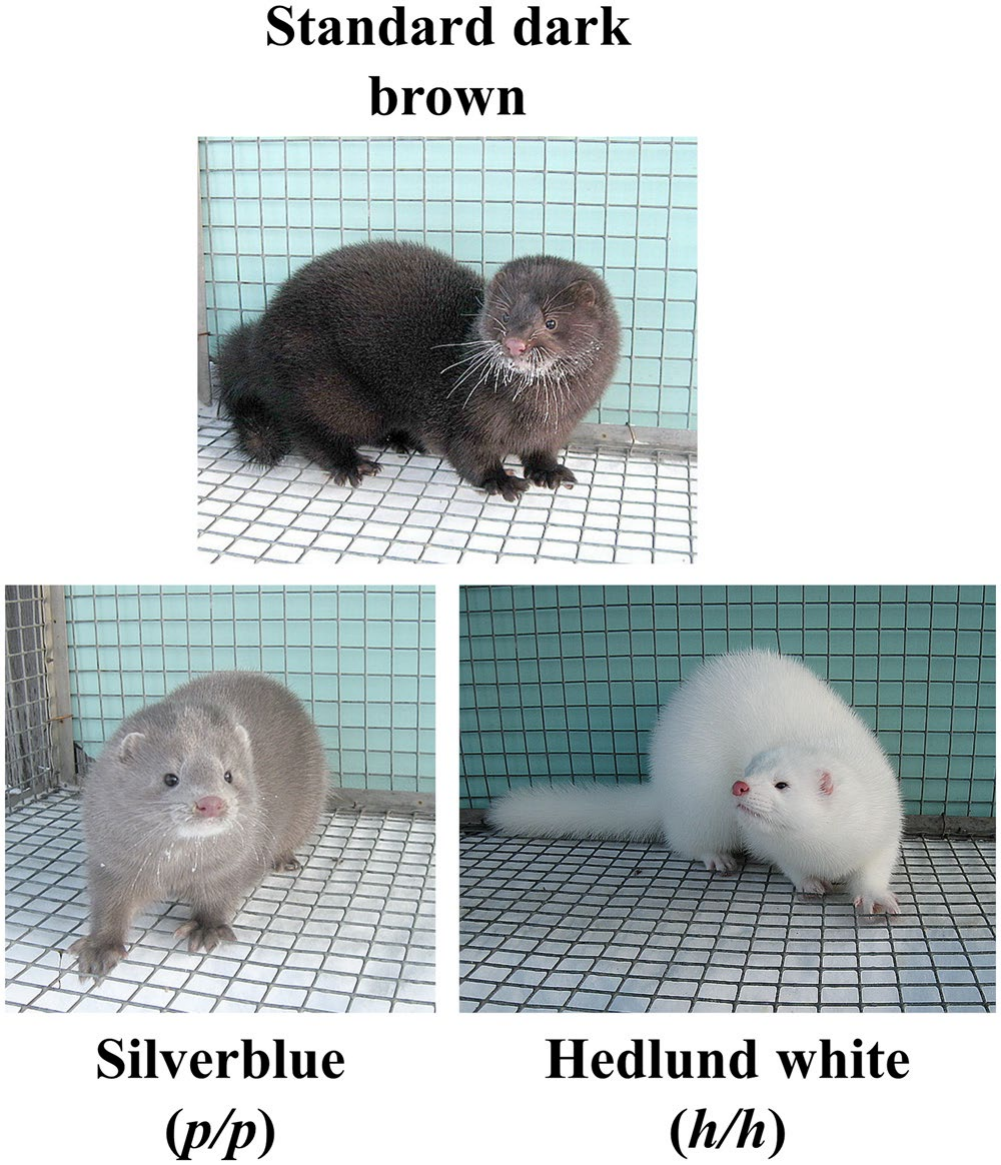
American minks of standard dark brown, Silverblue (*p/p*), and Hedlund white (*h/h*) phenotypes.

American mink is the most popular species in the global fur industry, accounting for 80% of international trade in unprocessed fur^1^. Mink’s fur quality is extremely high, and there is a wide range of colour variation derived from over a century of artificial selection on the original wild tawny brown phenotype (Fig. 1). To date, over 35 mutations affecting fur colour are known, and their combinations have resulted in the creation of over 100 forms^2^.

The first mink fur-colour mutant was described in 1931^2,3^, inherited as a Mendelian autosomal recessive trait and characterized by a Silverblue shade of the coat (Fig. 1). The mutation was originally named *platinum* due to similarity with an existing phenotype in foxes. Subsequently, the name was changed to Silverblue, although the mutation is still referred to as *p*^2^. This coat colour rose in popularity for the next 80 years, until it became one of the most common mutations in the mink fur industry. Furthermore, Silverblue is used in combination with other mutations to generate popular fur colours, such as violet (*m/m a/a p/p*), sapphire (*a/a p/p*), and pearl (*k/k p/p* or *k/k a/a p/p*)^2^.

Another widely used mutation is Hedlund white, which generates an albino-like white coat (Fig. 1)^4^. This phenotype is the result of a recessive mutation (*h*) with pleiotropic and codominance effects: homozygous (*h/h*) minks are completely white with dark or blue eyes and deaf, while heterozygous animals (*h/+*) are piebald with no hearing defects^4^.

In this study, we investigated the genetic mechanism determining both coat phenotypes through whole-genome sequencing of minks with Silverblue (*p/p*), and Hedlund white (*h/h*) and standard dark brown phenotypes.

## Results

This study, to the best of our knowledge, is the first to perform whole genome sequencing of American minks with three distinct fur colours. The average genome coverage was x5–10 (Supplementary Table 1).

### Silverblue fur colour is a result of splice donor site mutation of *MLPH* gene

We identified 187182 common homozygous variations in three Silverblue minks that were not in homozygous state in wild-type. Among 58 selected variations with putative “HIGH” impact, we found a single nucleotide variation (GL896909.1:662639 G/A (MLPH C.901 + 1 G > A), hereinafter referred to as *MLPH*^*p*^) at the splice donor site of melanophilin (*MLPH*) that potentially resulted in loss of function.

**Figure 2:**
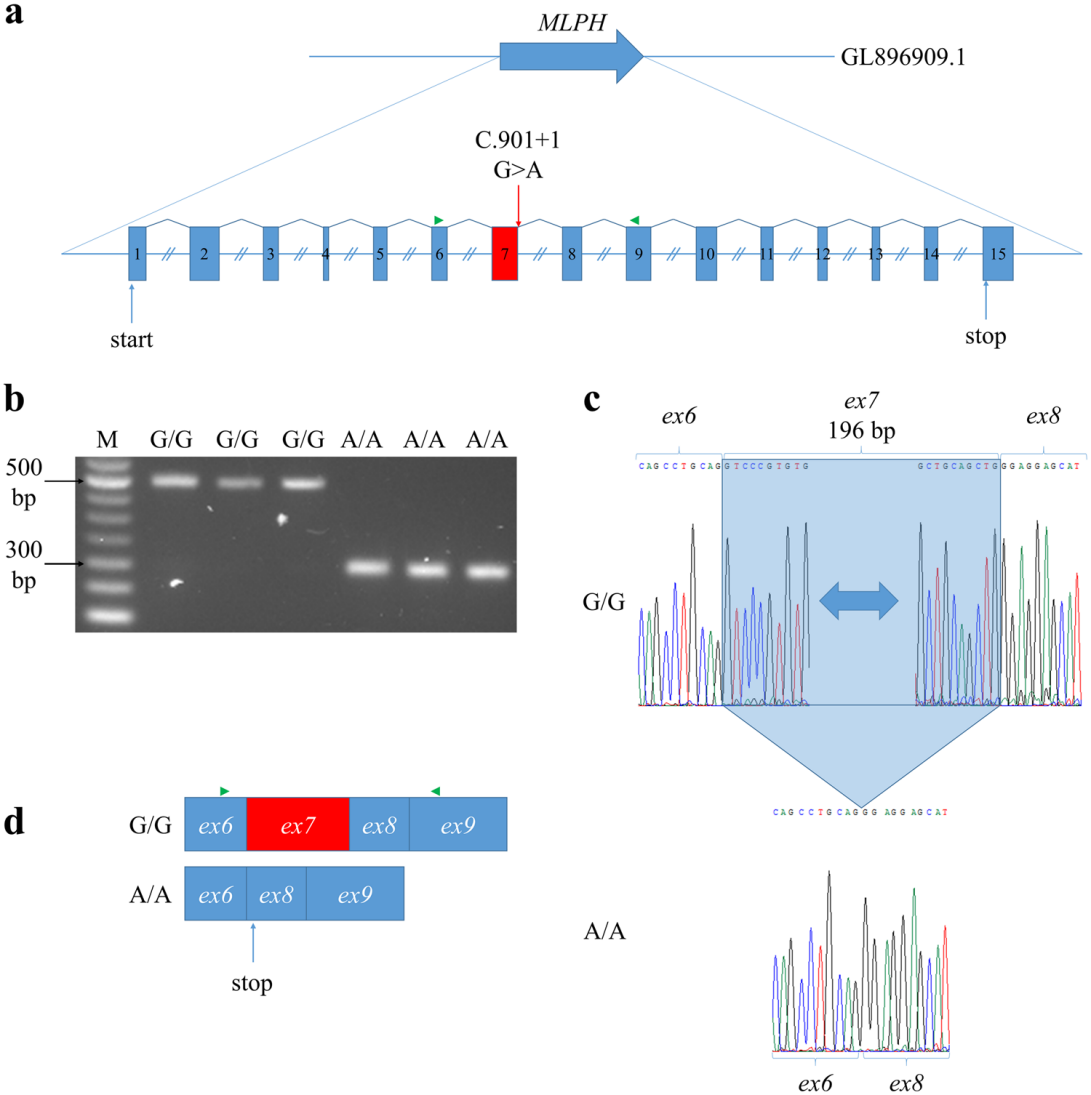
Effects of *MLPH*^*p*^ mutation on *MLPH* transcripts. (**a**) Structure of *MLPH* gene. Red box indicates exon 7. Green triangle indicates primers used for RT-PCR. Equal introns sizes are shown for simplification. (**b**) Agarose gel electrophoresis of *MLPH* cDNA exons 6–9. M – 50 bp DNA Ladder (NEB, USA). (**c**) An electrophoregram of Sanger sequencing for *MLPH* cDNA exons 6–9. Blue frame is exon 7 deleted in Silverblue (*p/p*) minks with homozygous *MLPH*^*p*^ mutation. (**d**) Effects of *MLPH*^*p*^ mutation on *MLPH* transcripts. Green triangle indicates primers used for RT-PCR.

Reverse transcription polymerase chain reaction (RT-PCR) was performed to confirm the predicted effect of homozygous *MLPH*^*p*^ mutation on the constitutive splicing donor site of *MLPH* exon 7. Sequencing of the cDNA region encompassing exons 6–9 revealed the complete loss of exon 7 in Silverblue (*p/p*) minks (Fig. 2).

We found that mutation *MLPH*^*p*^ was homozygous in all tested Silverblue (*p/p*) minks from two unrelated populations (Novosibirsk and Tver mink populations). Moreover, wild-type minks, as well as minks with other colour coats, are not homozygous for this mutation (Table 1). This mutation was also homozygous in all minks possessing the Silverblue allele *p*: pearl (*k/k p/p*), violet (*m/m a/a p/p*), and shadow silverblue minks (*S*^*H*^*/ + p/p*) (Table 1). These data suggest that mutation *MLPH*^*p*^ has a causative link to the Silverblue coat phenotype.

**Table 1:** Results of MLPH^p^ genotyping in American mink.

Coat colour	Population	Genotype			
		*GG*	*GA*	*AA*	*∑*
Standard dark brown	Novosibirsk	10	2	**0**	12
*k/k*		0	2	**0**	2
*b/b*		1	1	**0**	2
*m/m*		1	0	**0**	1
*a/a m/m*		1	0	**0**	1
*p/p*		0	0	**7**	7
*p/p*	Tver	0	0	**10**	10
*k/k p/p*	Novosibirsk	0	0	**2**	2
*m/m a/a p/p*		0	0	**1**	1
*S* ^*H*^ */ + p/p*		0	0	**2**	2

### Hedlund white fur colour is a result of splice donor site mutation of *MITF* gene

Based on genomic data, we identified 34736 homozygous genetic variations common across three Hedlund white minks, but not homozygous in both standard dark brown and Silverblue genomes. We found no potentially causative variants in selected homozygous variations across coding gene regions. Based on recently described associations between microphthalmia-associated transcription factor (*MITF*) locus and the Hedlund phenotype^4^, we analysed all variations in the *MITF* genomic region (Supplementary 1).

**Figure 3:**
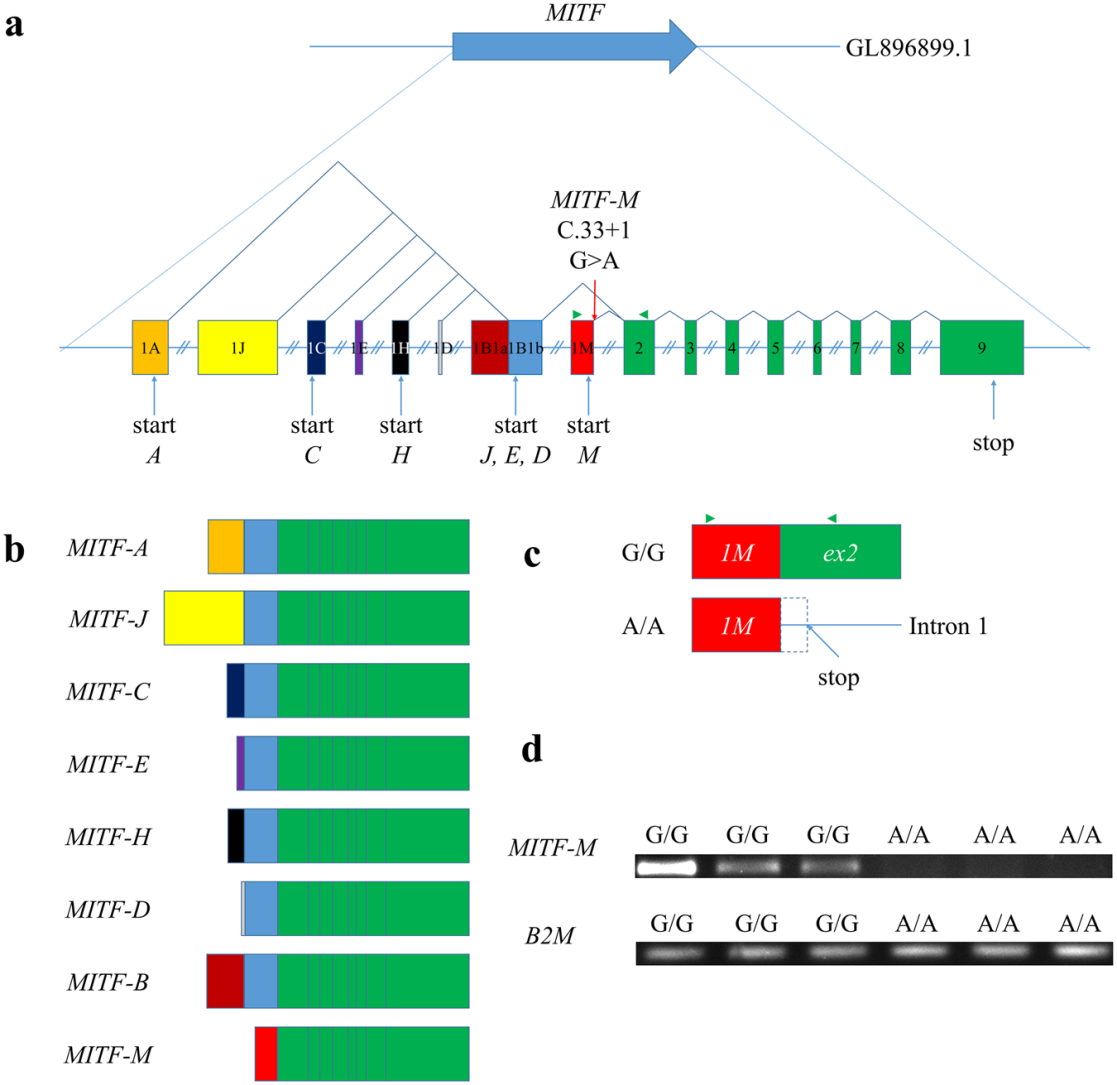
Effects of *MITF*^*h*^ mutation on transcripts of the *MITF* M-isoform. (**a**) Structure of mink *MITF* gene. Coloured boxes indicated exons 1 A (orange), 1 J (yellow), 1 C (dark blue), 1E (purple), 1 H (black), 1D (grey), 1B1a (brown), 1B1b (blue), 1 M (red) Green boxes indicate exons 2–9, common to all isoforms. Exons 1 A, 1 J, 1 C, 1E, 1 H, 1D, 1B1a and 1 M were predicted *in silico*. Green triangle indicates primers used for RT-PCR. Equal introns sizes are shown for simplification. (**b**) Structure of *in silico* predicted mink *MITF* isoforms (expression of *MITF-M* mRNA was confirmed with RT-PCR). Each isoform, except M, has a unique promoter and a first exon followed by 1B1b and 2–9 exons. The M-isoform is specific to melanocytes and melanoma cells, it does not include exon 1B1b. (**c**) Effect of *MITF*^*h*^ mutation on *MITF-M* transcript. This mutation potentially retains the first intron in cDNA and introduces a stop codon after position 51 of the intron (indicated as dotted box). The end product is a truncated 29 polypeptide containing only the first 11 amino acids of MITF-M. Green triangle indicates primers used for RT-PCR. (**d**) Agarose gel electrophoresis of *MITF-M* cDNA 1M-2 exons and *B2M* cDNA 1–2 exons. No *MITF-M* cDNA 1–2 exons were observed in the cortex of Hedlund white (*h/h*) minks, which were homozygous for this mutation.

Mammalian *MITF* gene contains multiple alternative promoters and consecutive first exons, followed by common downstream exons, thereby generating at least eight isoforms with different N-termini (Fig. 3). Expression patterns of different isoforms range from widely expressed to tissue-specific^5,6^. Only one *MITF* transcript was found in both annotated ferret genome (Ensemble MusPutFur1.0.86) and mink transcriptome^7^. By using previously published *MITF* transcripts sequences for human, mice and dog *MITF* gene isoforms^8–10^, we perform *in silico* prediction of other *MITF* transcripts, and found eight *MITF* gene isoforms (A, J, C, E, H, D, B and M) in ferret genome (Supplementary 2). Next, we repeated SnpEff annotations and effect predictions of selected genetic variations in Hedlund white mink genome with all six new *MITF* isoforms. As a result, we identified single nucleotide variation (GL896899.1:18635719 G/A (MITF-M C.33 + 1 G > A), hereinafter referred to as *MITF*^*h*^) at the splice donor site of the 1 M exon in melanocyte-specific MITF isoform (MITF-M). This isoform is expressed from the most downstream promoter of *MITF* in melanocytes, melanoma cells^6^ and brain^11^. The isoform MITF-M was a crucial regulator of melanoblast migration from the neural tube, as well as survival and differentiation of melanoblasts into melanocytes^5,8^.

The mutation *MITF*^*h*^ potentially leads to a stop codon after position 51 of the first MITF-M intron, resulting in a truncated 29 amino acid product, that contains only the first 11 amino acid of MITF-M. The mutation was homozygous in all tested Hedlund white minks from the two unrelated test populations, but not in minks with other coat colour phenotypes (Table 2).

**Table 2:** Results of MITF^h^ genotyping in American mink.

Coat colour	Population	Genotype			
		*GG*	*GA*	*AA*	*∑*
Standard dark brown	Novosibirsk	8	1	**0**	9
*k/k*		2	0	**0**	2
*b/b*		2	0	**0**	2
*m/m*		0	1	**0**	1
*a/a m/m*		1	0	**0**	1
*p/p*		5	1	**0**	6
*k/k p/p*		2	0	**0**	2
*m/m a/a p/p*		1	0	**0**	1
*S* ^*H*^ */ + p/p*		2	0	**0**	2
*h/h*		0	0	**12**	12
*h/h*	Tver	0	0	**4**	4

MITF-M isoforms are unlikely to be detected in Hedlund-white mink skin cells, due to the previously demonstrated disturbance of melanocyte migration in their skin^4^. Nevertheless, MITF-M isoform was found to be expressed in mouse brain tissues^11^. By using RT-PCR we detect the *MITF-M* transcript specific region (encompassing splicing site exons 1 M and 2) in cortex of standard dark brown and Silverblue (*p/p*) minks. However, this region was absent in transcripts from cortices of Hedlund white (*h/h*) minks, which were homozygous for *MITF*^*h*^ (Fig. 3). Therefore, we suggest that the mutation violates the structure of MITF-M mRNA, resulted in the absence of functional MITF-M mRNA and protein.

## Discussion

In the present study, we described mutations in splice donor sites of American-mink *MLPH* and *MITF* genes. These mutations (MLPH C.901 + 1 G > A and MITF-M C.33 + 1 G > A) respectively cause the commercially valuable Silverblue and Hedlund white phenotypes.

**Figure 4:**
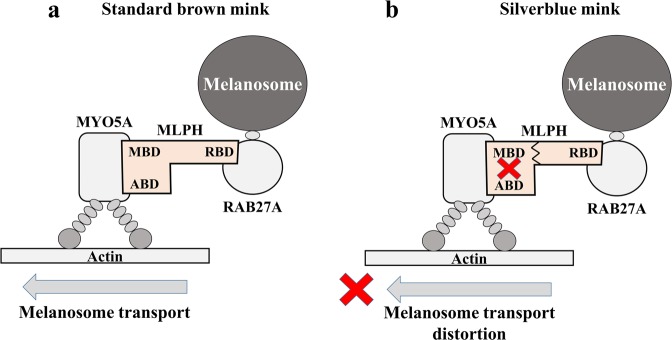
Scheme of tripartite complex (RAB27A-MLPH-MYO5A) in melanosome intracellular trafficking in standard dark brown (**a**) and Silverblue (**b**) minks. RBD - RAB27-binding domain; MBD - MYO5A-binding domain; ABD - actin-binding domain.

The *MLPH* gene encodes melanophilin, a Rab effector protein involved in melanosome transport. It acts as a linker between melanosome-bound RAB27A and the motor protein MYO5A in melanosome trailing. MLPH contains N-terminal RAB27-binding domain (RBD), a medial MYO5A-binding domain (MBD), and C-terminal actin-binding domain (ABD), which enhances MLPH interaction with MYO5A^12^. The C-terminal domain can also interact with microtubule-associated protein EB1^12,13^. A tripartite complex (RAB27A-MLPH-MYO5A) is among the most important elements of mature melanosome intracellular trafficking^12^ (Fig. 4a). In Silverblue minks, complete loss of *MLPH* exon 7 leads to a frame shift and a premature stop-codon at amino-acid position 308. This truncated protein lacks the C-terminal actin-binding domain and exon F binding domain (EFBD), both are parts of the MYO5A-binding domain. Previous studies demonstrated that EFBD region of MBD is necessary for MYO5A recruitment, while ABD is required for the MYO5A-MLPH association^12^. Dysfunction in MYO5A recruitment disturbs the transport of mature melanosomes to actin-rich dendritic tips of melanocytes, here, melanosomes are passed to the nearest keratinocytes (Fig. 4b). Disturbs of this process eventually dilute coat colour^14^.

We note that the *MLPH* gene was previously associated with mink Silverblue fur colour^15,16^. However, there was no convincing evidence for the molecular phenotype determining Silverblue coat colour in American mink. In this study we identify that all tested Silverblue (*p/p*), pearl (*k/k p/p*), violet (*a/a m/m p/p*) and shadow silverblue (*S*^*H*^*/ + p/p*) minks possessed homozygous *MLPH*^*p*^ mutation and confirmed that it has deleterious effect on *MLPH* mRNA. This observation implies that mutation in the *MLPH* splice donor site is causative link to the Silverblue coat phenotype.

*MITF* gene, especially its melanocyte-specific isoform MITF-M, is well known to be critical for the development of neural-crest-derived melanocytes. *MITF* gene mutations cause abnormal depigmentation of hair and skin, sometimes associated with total or partial deafness. Such mutations were previously described in mouse^5,17^, Syrian hamster^18^, cattle^19^, dog^20^, and human (Tietz albinism-deafness syndrome: OMIM#103500 and Waardenburg syndrome, type 2 A: OMIM#193510)^21^. A strong association was previously indicated between the locus containing *MITF* and the Hedlund phenotype in mink. However, the earlier study did not reveal any mutations in coding and intron flanking sequences of the MITF-M in Hedlund white minks^4^. We suggest that the identified mutation GL896899.1:18635719 G/A in *MITF* is causative for the Hedlund white phenotype.

To date, only three mink colour genes have been mapped: *TYR* (albino and Himalayan breeds)^22,23^, *LYST* (Aleutian)^24^, and *TYRP1* (American palomino)^25^. Our present study adds *MLPH* and *MITF* to this list, providing valuable data that can contribute to improving global mink fur production through selective breeding programs.

## Methods

Silverblue (*p/p*, 7 individuals), Hedlund white (*h/h*, 12 individuals), and standard dark brown (12 individuals) farm-bred American minks were maintained in the Experimental Fur Farm of the Institute of Cytology and Genetics, Siberian Branch of the Russian Academy of Sciences (Novosibirsk mink population), were used in the study. The study protocols were approved by the local Ethics Committee of the Institute of Cytology and Genetics. Minks were euthanized by carbon dioxide asphyxiation followed by decapitation according to the published protocol^26^ and the institutional guidelines on animal welfare. Cortices, testis, and muscles were rapidly dissected and frozen in liquid nitrogen, then stored at −70 °C until DNA and RNA extraction. We also collected samples from pearl (*k/k p/p*, 2 individuals), violet (*m/m a/a p/p*, 1 individual), shadow silverblue (*S*^*H*^*/ + p/p*, 2 individuals), royal pastel (*b/b*, 2 individuals), American palomino (*k/k*, 2 individuals), moyle (*m/m*, 1 individual), and lavender (*a/a m/m*, 1 individual) minks (Novosibirsk mink population).

In addition, the sample collection from farm-bred American minks of Silverblue (*p/p*, 10 individuals) and Hedlund white (*h/h*, 4 individuals) coat colours from «Mermeriny» fur farm, Tver region, Russia (Tver mink population) was used in this study. Tver mink population is unrelated to Novosibirsk one.

Genomic DNA from mink tissues was extracted using QIAGEN Mini Spin Columns, following manufacturer protocol (QIAGEN, Germany). Library preparation was performed with the TruSeq DNA HT Sample Prep Kit (Illumina, USA), with slight modifications to manufacturer protocol. In brief, between 500 ng and 1 μg of genomic DNA was fragmented to a mean target size of approximately 300–400 bp using sonication in a Covaris S2 (Covaris, USA). Fragmented DNA was end-repaired, A-tailed, and indexed using TruSeq Illumina adapters with overhang-T followed by DNA purification from the reaction mixture with MinElute PCR Purification and QIAquick PCR Purification Kits (QIAGEN). Purified DNA with ligated adapters was used for size selection in 2% agarose SizeSelect E-Gel (Invitrogene, USA). Fragments of 400–500 bp were then isolated from agarose with Gel Extraction Kit (QIAGEN) and used for library amplification. Enriched libraries were purified using QIAquick PCR Purification Kits (QIAGEN), followed by 2% agarose SizeSelect E-Gel (Invitrogene, USA) electrophoresis to remove primer dimers. Library validation was performed with an Agilent 2100 Bioanalyzer with DNA High Sensitivity chip (Agilent, USA), and quantified with qPCR using a KAPA Library Quantification Illumina Kit protocol (KAPA Biosystems, USA). Paired-end libraries were sequenced in 2 × 101 cycles with the Illumina TruSeq SBS v3 kit (Illumina) on a HiSeq 2000/2500 sequencer (Illumina) at the Vavilov Institute of General Genetics RAS (Moscow, Russia).

Resulting reads were mapped to the ferret (*Mustela putorius furo*) genome (MusPutFur1.0) using a BWA-MEM algorithm (bwa v.0.7.13-r112)^27^. Duplicate reads were detected with the MarkDuplicates algorithm from picard-tools v.1.133 (broadinstitute.github.io/picard) and excluded from the further analysis.

Genetic variants in sequenced mink genomes were predicted in Freebayes v1.0.2–29-g41c1313^28^, with *min-base-quality* and *min-mapping-quality* settings of 3 and 1, respectively. Genetic variations were filtered (QUAL ≥ 1 RPR > 0 and RPL > 0 settings) with vcflib software (github.com/vcflib). Insertions and deletions (InDels) were then realigned against the reference ferret genome with bcftools 1.3.1^29^. Annotation and effect prediction of selected variants were performed in SnpEff version 4.2^30^, using the ferret genome annotation (Ensemble MusPutFur1.0.86).

To detect the genetic factor underlying the Silverblue phenotype, we selected common homozygous variants in three *p/p* minks that are not homozygous in standard dark brown wild-type animals. Hedlund white minks were not used in this analysis because the *h* mutation has an epistatic effect, causing a lack of melanocytes, Hedlund white^4^ and therefore unable to express any fur colours. Similarly, we separately selected common homozygous variants in three Hedlund white (*h/h*) genomes that are not homozygous in the other two colour phenotypes.

We performed Sanger sequencing to validate mutations. Primers for PCR amplification were designed in Primer3 software (Table 3), and PCR was performed with GenPack PCR Core (Isogen, Russia). Resultant amplicons were cleaned with a Cleanup Standard Kit (Evrogen, Russia) and processed with the BigDye® Terminator v3.1 Cycle Sequencing Kit (Applied Biosystems, USA), following manufacturer protocol. Probes were purified using a DyeEx 2.0 Spin Kit (QIAGEN) and sequenced in a 3730xl DNA Analyzer (Applied Biosystems).

**Table 3:** Primer sequences used for cDNA and gDNA amplification.

Primer name	Primer sequence	Expected amplicon size (bp)	Annealing t (°C)
cDNA MLPH ex 6–9 F	TTTGAGGCTGACTCTGACGA	486	60.0
cDNA MLPH ex 6–9 R	CCTCCTGAGGGTCTCCTCTT		
gDNA MLPH ex 7 F	CCTCCAGAAGAGCAGATGG	335	58.0
gDNA MLPH ex 7 R	GAGCTATTGATGCTGGGACT		
cDNA MITF ex 1M-2 F	CTTCTCTATGCCCGTCAGTC	241	57.5
cDNA MITF ex 1M-2 R	GGTTGGCATGTTTATTTGCT		
gDNA MITF ex 1 M F	CTTCTCTATGCCCGTCAGTC	368	58.0
gDNA MITF ex 1 M R	GAACAGGAGCTGATGGAGAG		
сDNA B2M ex 1–2 F	TTCTCTGGACGTTGGTCTTC	236	58.0
сDNA B2M ex 1–2 R	GAAACTCCAGTCCTTGCTGA		

Effects of *MITF* and *MLPH* mutations on splicing were validated using RT-PCR. Total RNA was extracted from cortex and testis using RNeasy Mini Spin Columns, following manufacturer protocol (QIAGEN). Extracted RNA was then treated with RNase-Free DNase I (Epicentre, USA), then assayed for quantity and quality with NanoDrop One-C (Thermo Scientific, USA). All RNA samples were kept at −80 °C. First-strand cDNA synthesis was performed using 300 ng of RNA with a High-Capacity cDNA Reverse Transcription Kit (Applied Biosystems).

## Supplementary information

Supplementary 1.

Supplementary info
