# Supplementary material for: Genome analysis identifies the mutant genes for common industrial Silverblue and Hedlund white coat colours in American mink
Source: Sci Rep. 2019 Mar 14;9:4581. doi: 10.1038/s41598-019-40918-7 (PMC6418256; doi:10.1038/s41598-019-40918-7)

Genome analysis identifies the mutant genes for common industrial Silverblue and Hedlund white coat colours in American mink

Andrey D. Manakhov[1, 2, 3] (orcid: 0000-0002-5163-8747)

Tatiana V. Andreeva[1, 3]

Oleg V. Trapezov[4,5]

Nikolay A. Kolchanov[2] (orcid: 0000-0001-6800-8787)

Evgeny I. Rogaev[1, 3, 6, 7] *

1) Department of Genomics and Human Genetics, Vavilov Institute of General Genetics, Russian Academy of Sciences, Moscow, 119991, Russia
2) Systems Biology Department, Institute of Cytology and Genetics, Siberian Branch of the Russian Academy of Sciences, Novosibirsk, 630090, Russia
3) Center for Genetics and Genetic Technologies, Faculty of Biology, Lomonosov Moscow State University, Moscow, 119234, Russia
4) Department of Animals and Human Genetics, Institute of Cytology and Genetics, Siberian Branch of the Russian Academy of Sciences, Novosibirsk, 630090, Russia
5) Novosibirsk State University, Novosibirsk, 630090, Russia
6) Center for Brain Neurobiology and Neurogenetics, Institute of Cytology and Genetics, Siberian Branch of the Russian Academy of Sciences, Novosibirsk, 630090, Russia
7) Department of Psychiatry, Brudnick Neuropsychiatric Research Institute, University of Massachusetts Medical School, Worcester, MA 01604, USA

* Corresponding author (rogaev@vigg.ru; Vavilov Institute of General Genetics, Russian Academy of Sciences, Moscow, 119333, Russia; tel: 8 (499) 135-50-61)

Supplementary Table 1. Results of sequencing American mink genomes. Statistics were calculated with samtools[1], picard, and GATK[2] software, ferret genome (MusPutFur1.0) was used as reference.

**Sample**	**Colour**	**N of reads**	**Mapped %**	**Duplicates %**	**Coverage***
mink_3-261	Standard dark brown	266,270,413	96,18	4,41	10,04
mink_3-247	Standard dark brown	257,506,707	97,64	3,06	9,78
mink_3-265	Standard dark brown	235,071,238	96,77	2,56	9,14
mink_1-663	*p/p*	177,249,440	95,38	11,11	6,63
mink_0-329	*p/p*	170,946,890	97,18	3,43	6,54
mink_9-431	*p/p*	151,699,053	95,36	4,27	5,57
mink_1-679	*h/h*	140,973,939	95,14	4,38	5,29
mink_1-725	*h/h*	139,370,841	95,82	3,57	5,26
mink_1-681	*h/h*	136,213,566	94,09	3,27	5,02

1. Li, H. *et al.* The Sequence Alignment/Map format and SAMtools. *Bioinformatics* **25,** 2078–2079 (2009).

2. McKenna, A. *et al.* The genome analysis toolkit: A MapReduce framework for analyzing next-generation DNA sequencing data. *Genome Res.* **20,** 1297–1303 (2010).

Supplementary 2. Ferret *MITF* isoforms.

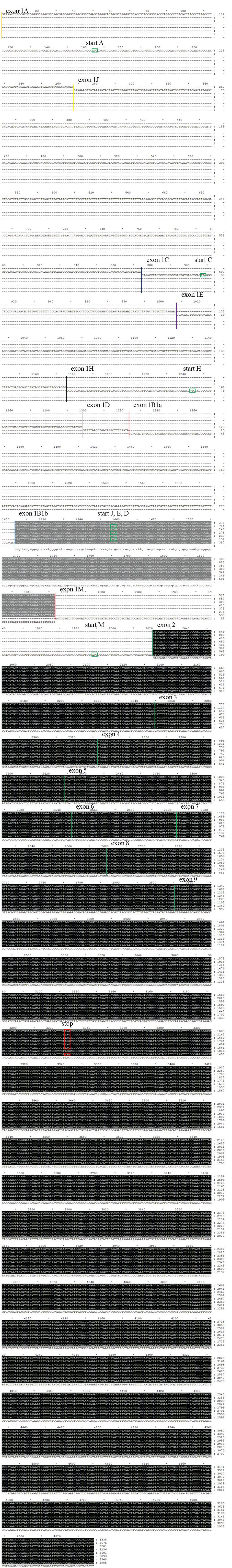

Supplementary 3. Original agarose gel electrophoresis of *MLPH* cDNA exons 6–9. M – 50 bp DNA Ladder (NEB, USA). Genotypes for GL896909.1:662639 G/A (MLPH C.901+1 G>A) mutation are presented.

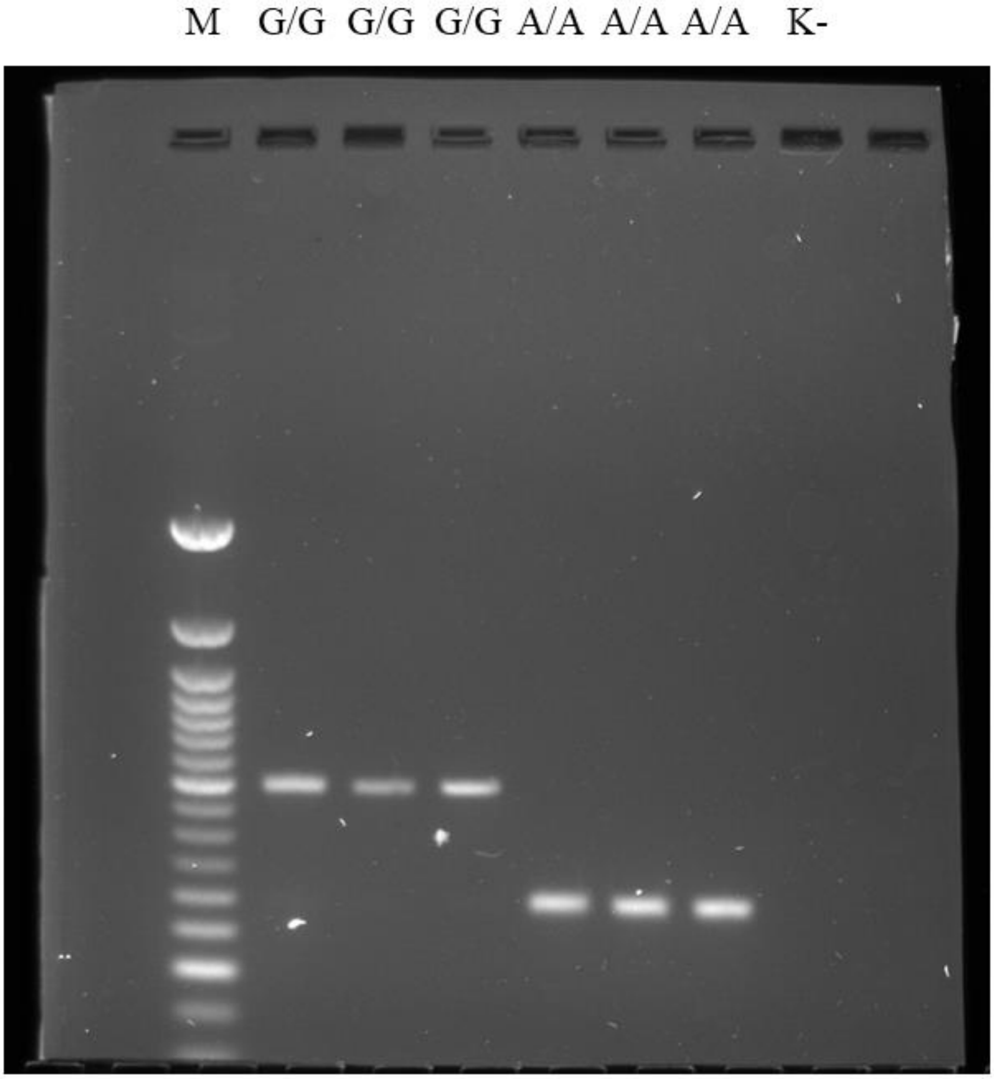

Supplementary 4. Original agarose gel electrophoresis of *MITF-M* cDNA 1M-2 exons and *B2M* cDNA 1–2 exons. No *MITF-M* cDNA 1–2 exons were observed in the cortex of Hedlund white (*h/h*) minks, which were homozygous for this mutation. M – DNA ladder 100 bp + 50 bp (SibEnzyme, Russia). Genotypes for GL896899.1:18635719 G/A (MITF-M C.33+1 G>A) mutation are presented.

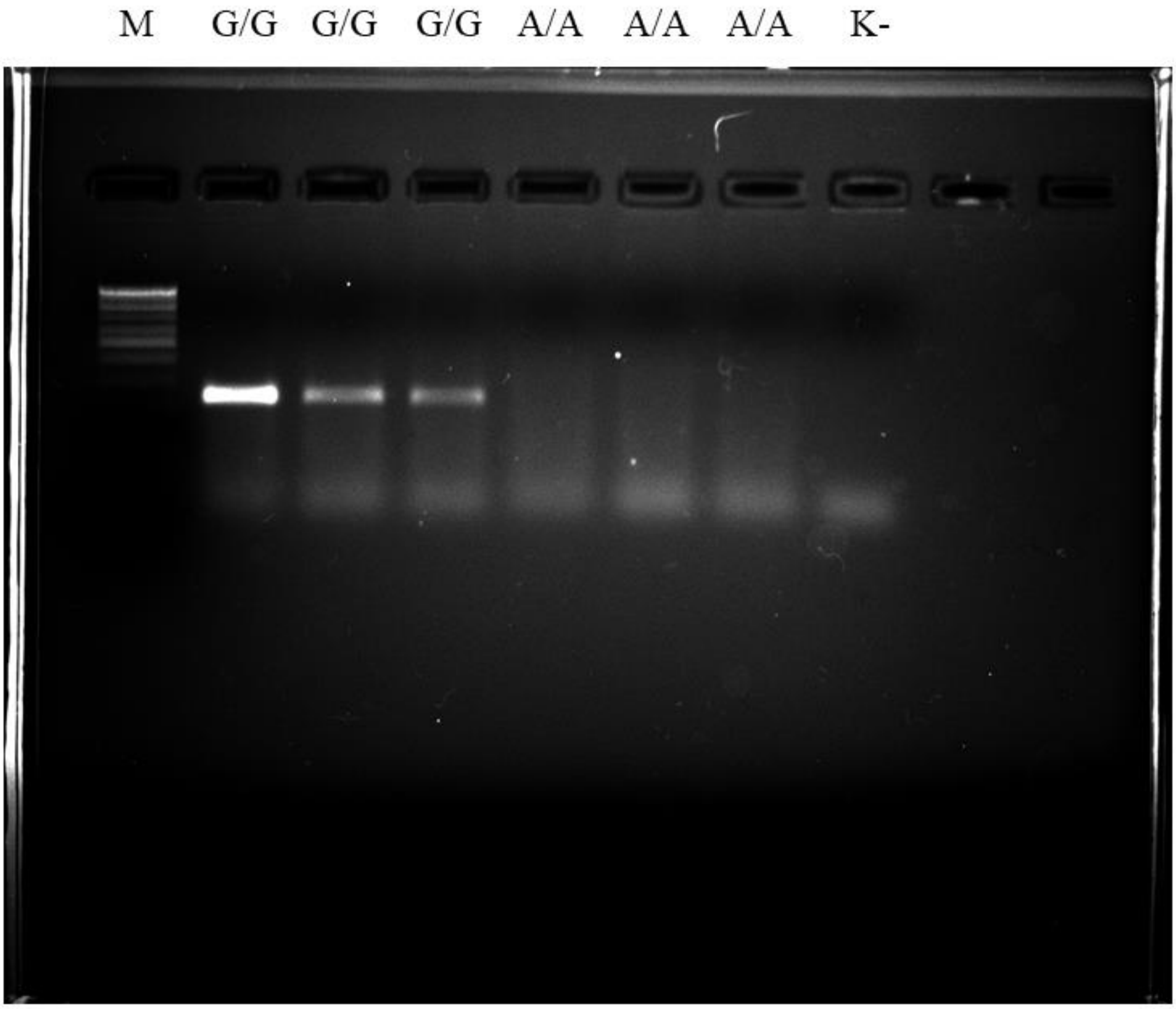

Supplementary 5. Original agarose gel electrophoresis of *B2M* cDNA 1–2 exons. 50 bp DNA Ladder (NEB). Genotypes for GL896899.1:18635719 G/A (MITF-M C.33+1 G>A) mutation are presented.

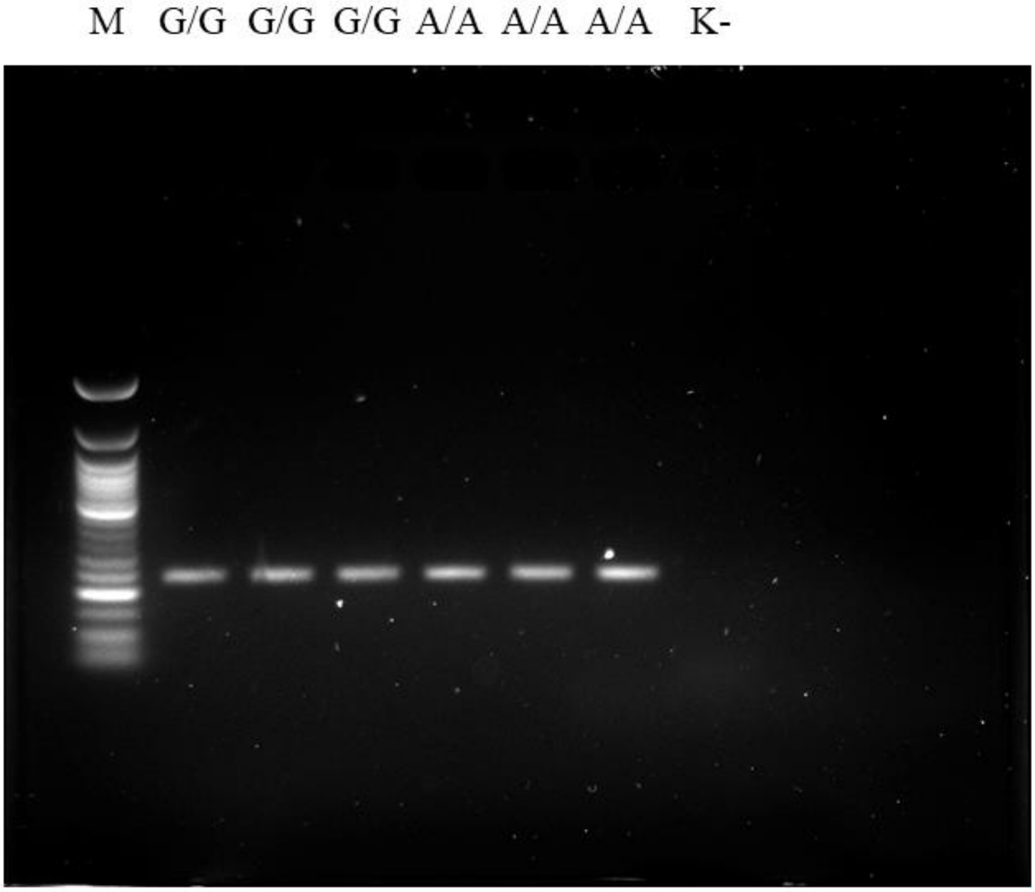

Supplement: Supplementary file 2 — Supplementary info [file 41598_2019_40918_MOESM2_ESM.pdf]
